# Crystal structures of *N*-(3-fluoro­benzo­yl)benzene­sulfonamide and *N*-(3-fluoro­benzo­yl)-4-methyl­benzene­sulfonamide

**DOI:** 10.1107/S2056989016003248

**Published:** 2016-03-02

**Authors:** P. A. Suchetan, S. Naveen, N. K. Lokanath, H. N. Lakshmikantha, K. S. Srivishnu, G. M. Supriya

**Affiliations:** a Department of Chemistry, University College of Science, Tumkur University, Tumkur 572 103, India; b Institution of Excellence, University of Mysore, Manasagangotri, Mysuru-6, India; c Department of Physics, University of Mysore, Manasagangotri, Mysuru-6, India; d University College of Science, Tumkur, India

**Keywords:** crystal structure, *N*-(aryl­sulfon­yl)aryl­amides, N—H⋯O hydrogen bonds, C—H⋯O inter­actions, C—H⋯π inter­actions

## Abstract

The crystal structures of two *N*-(aryl­sulfon­yl)aryl­amides, namely *N*-(3-fluoro­benzo­yl)benzene­sulfonamide, C_13_H_10_FNO_3_S, (I), and *N*-(3-fluoro­benzo­yl)-4-methyl­benzene­sulfonamide, C_14_H_12_FNO_3_S, (II), are described and compared with related structures. The dihedral angle between the benzene rings is 82.73 (10)° in (I) compared to 72.60 (12)° in (II). In the crystal of (I), the mol­ecules are linked by C—H⋯O and C—H⋯π inter­actions, resulting in a three-dimensional grid-like architecture, while C—H⋯O inter­actions lead to one-dimensional ribbons in (II). The crystals of both (I) and (II) feature strong but non-structure-directing N—H⋯O hydrogen bonds with *R*_2_^2^(8) ring motifs. The structure of (I) also features π–π stacking inter­actions.

## Abstract 2

In the title compounds, N—H⋯O hydrogen bonds lead to dimers; the dimers are linked by weak inter­actions into a three-dimensional network in one case and chains in the other.

## Chemical context   

*N*-(Aryl­sulfon­yl)aryl­amides have received much attention as they constitute an important class of drugs for Alzheimers disease (Hasegawa *et al.*, 2000 ▸), anti­bacterial inhibitors of tRNA synthetases (Banwell *et al.*, 2000 ▸), antagonists for angiotensin II (Chang *et al.*, 1994 ▸) and as leukotriene D4-receptors (Musser *et al.*, 1990 ▸). Further, *N*-(aryl­sulfon­yl)aryl­amides are known to be potent anti­tumour agents against a broad spectrum of human tumour xenografts (colon, lung, breast, ovary and prostate) in nude mice (Mader *et al.*, 2005 ▸). As part of our ongoing work on the synthesis and crystal structures of this class of compound (Gowda *et al.*, 2009*a* ▸,*b* ▸; Sreenivasa *et al.*, 2014 ▸; Suchetan *et al.*, 2010 ▸, 2012 ▸), compounds (I) and (II) were synthesized and their crystal structures were determined.
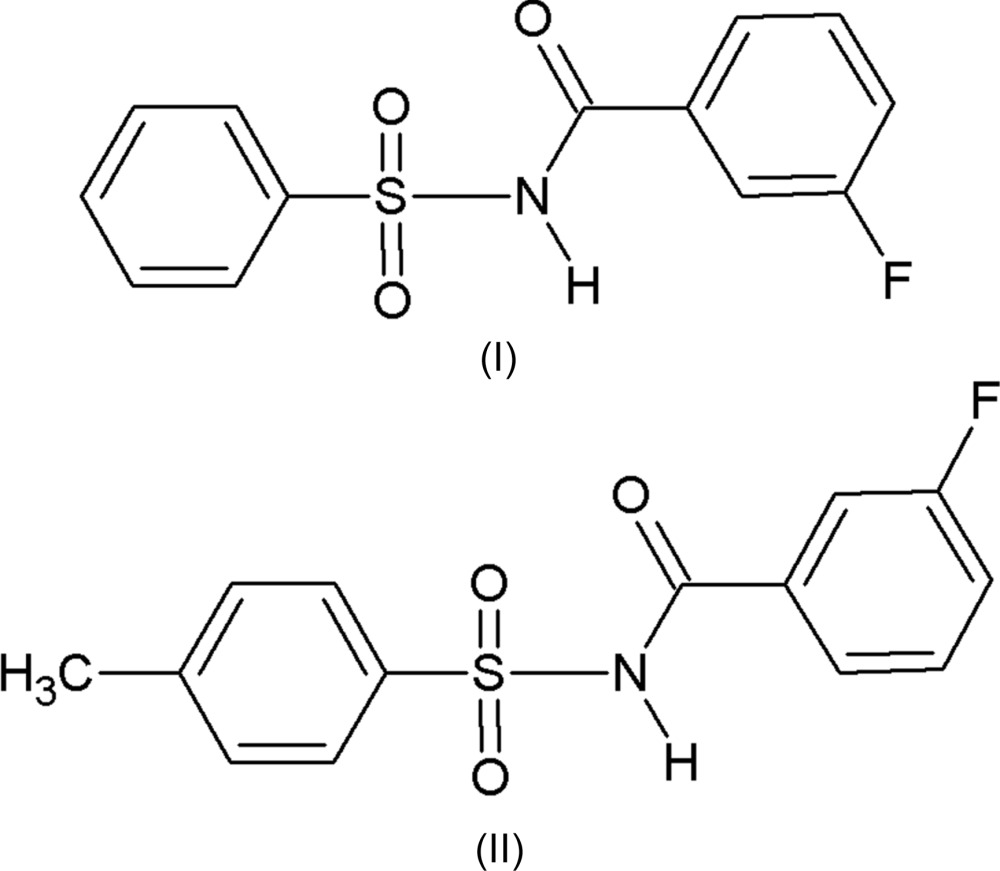


## Structural commentary   

**Figure 1:**
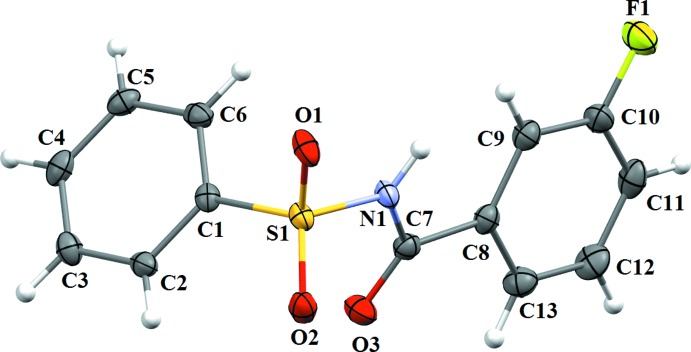
A view of the mol­ecular structure of (I), with displacement ellipsoids drawn at the 50% probability level.

**Figure 2:**
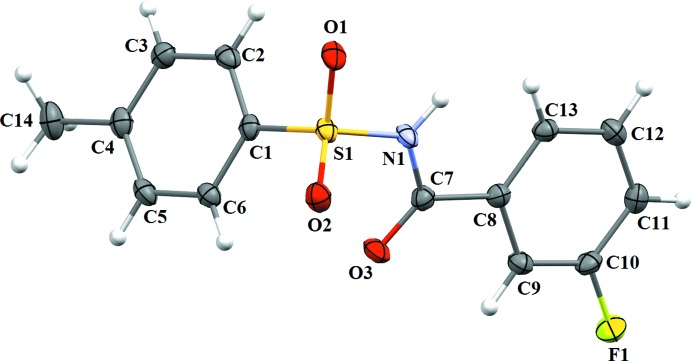
A view of the mol­ecular structure of (II), with displacement ellipsoids drawn at the 50% probability level.

The *meta*-fluoro substitution on the benzoyl ring of (I) (Fig. 1 ▸) is *syn* to the N—H bond in the central –C—SO_2_—N—C(=O)– segment. By contrast, in (II) (Fig. 2 ▸), the conformation of the N—H bond is *anti* with respect to the *meta*-fluoro substitution on the benzoyl ring. The dihedral angle between the benzene rings is 82.73 (10)° in (I), while, in (II) the value is slightly less [72.60 (12)°]. Further, in (I), the dihedral angle between the benzoic acid ring and the central C8—C7(O3)—N1—S1 segment is 16.54 (10)°, while that between the sulfonamide ring and the C7(O3)—N1—S1—C1 segment is 81.87 (12)°. The corresponding values in (II) are slightly less than those observed in (I), being 12.12 (12) and 57.58 (13)°, respectively.

## Supra­molecular features   

**Figure 3:**
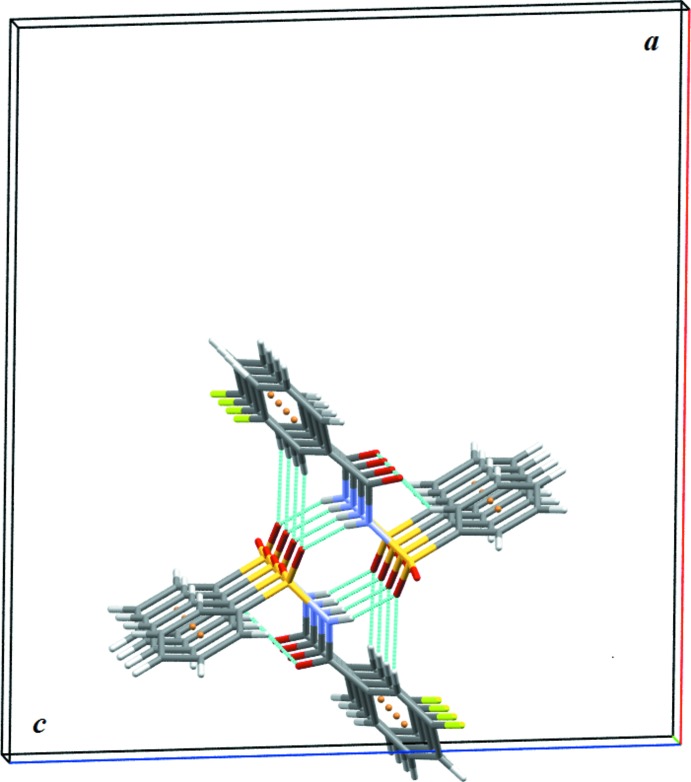
Crystal packing of (I), displaying N—H⋯O hydrogen bonds and C—H⋯O inter­actions, which result in columns along the *b* axis.

**Figure 4:**
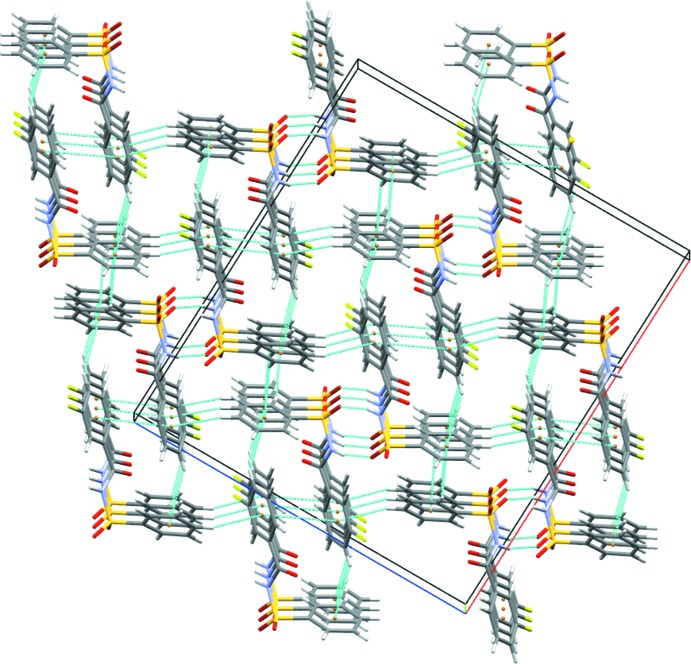
Three-dimensional grid-like architecture formed by various C—H⋯π_ar­yl_ inter­actions in (I).

The crystal structure of (I) features strong N1—H1⋯O1 hydrogen bonds (Table 1 ▸) that connect the mol­ecules into 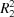
(8) dimers (Fig. 3 ▸). These dimers are further inter­connected by C9—H9⋯O1 inter­actions, forming 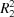
(14) ring motifs. C6—H6⋯O3 inter­actions connect these dimers into *C*7 chains, forming columns propagating along the *b*-axis direction (Fig. 3 ▸). In addition, C4—H4⋯π_ar­yl_ (π system of the fluoro­benzoyl ring) inter­actions link the mol­ecules into chains along the *c* axis. These chains are inter­connected *via* C2—H2⋯π_ar­yl_ (π system of the sulfonyl­benzene ring) and C11—H11⋯π_ar­yl_ (π system of the sulfonyl­benzene ring) inter­actions, forming a three-dimensional grid-like structure (Fig. 4 ▸). The crystal structure also features π–π (π system of the fluoro­benzoyl ring) stacking inter­actions. It is notable that the N—H⋯O hydrogen bonds present in the crystal structure of (I) has no structure-directing properties (leading only to dimers), while one of the C—H⋯O and the three C—H..π_ar­yl_ inter­actions have structure-directing characteristics.

**Figure 5:**
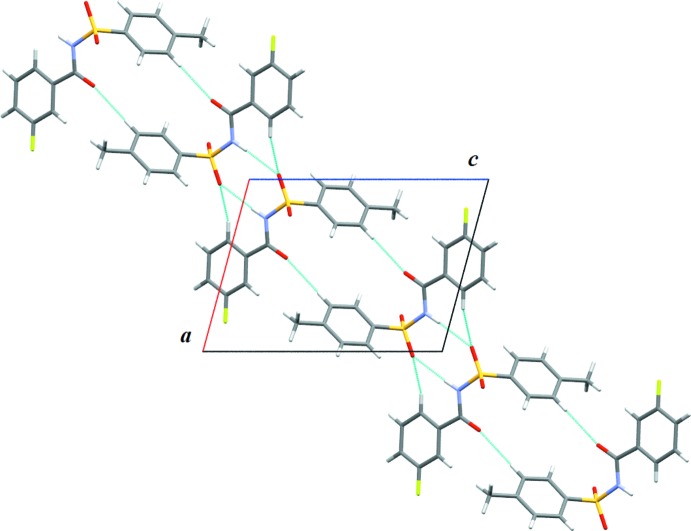
One-dimensional ribbons formed in the crystal structure of (II) *via* N—H⋯O dimeric pairs and various C—H⋯O dimeric pairs.

Similar to that observed in the crystal structure of (I), in (II) strong N1—H1⋯O1 hydrogen bonds (Table 2 ▸) result in the formation of 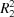
(8) dimers (Fig. 5 ▸). The mol­ecules constituting these dimers are inter­connected into 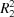
(14) ring motifs *via* C13—H13⋯O1 inter­actions, as observed in (I). Adjacent dimers are inter­connected *via* C5—H5⋯O3 inter­actions into 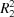
(16) rings, thus forming ribbons along the diagonal of the *ac* plane (Fig. 5 ▸). The overall supra­molecular architecture displayed in (II) is one-dimensional, in contrast to the three-dimensional architecture displayed in (I).

## Database survey   

The crystal structures of five related *N*-(aryl­sulfon­yl)aryl­amides, namely *N*-(benzo­yl)benzene­sulfonamide (III), *N*-(3-chloro­benzo­yl)benzene­sulfonamide (IV), *N*-(3-methyl­benzo­yl)benzene­sulfonamide (V), *N*-(benzo­yl)-4-methyl­benzene­sulfon­amide (VI) and *N*-(3-methyl­benzo­yl)-4-meth­ylbenzene­sulfonamide (VII) have previously been reported. A comparison of the dihedral angle between the two benzene rings in these closely related structures indicates that introducing a methyl substituent into the *para* position of the benzene­sulfonyl ring lowers the dihedral angle with compound (VII) being an exception. The dihedral angle values are 80.3 (1)° in (III) (Gowda *et al.*, 2009*a* ▸), 87.5 (1)° in (IV) (Gowda *et al.*, 2009*b* ▸), 83.3 (2), 84.4 (2) and 87.6 (2)° in the three mol­ecules of (V) (Suchetan *et al.*, 2012 ▸), 79.4 (1)° in (VI) (Suchetan *et al.*, 2010 ▸) and 89.6 (2)° in (VII) (Sreenivasa *et al.*, 2014 ▸). This effect is the same as that observed in the present two structures (I) and (II). Furthermore, in (I)–(VII) the conformation of the N—H bond in the central segment is *anti* to the *meta* substituent on the benzoyl ring in the presence of a methyl substituent either on the benzoyl ring or the benzene­sulfonyl ring. Otherwise, the conformation is *syn* as observed in (I) and (IV). A comparison of the crystal structures of (I) and (II) with those previously reported shows that fluoro substitution on the benzoyl ring appears to have a significant effect on the supra­molecular architecture, and also on the type and nature of the inter­molecular inter­actions displayed. For instance, in all the reported structures except (VII), the mol­ecules are linked into one-dimensional infinite *C*(4) chains *via* strong structure-directing N—H⋯O hydrogen bonds. The structures do not feature any other type of inter­actions. However, in (I) and (II), the N—H⋯O hydrogen bonds lead to dimers and, in addition, both of them feature other structure-directing inter­actions of the type C—H⋯O or C—H⋯π_ar­yl_. Furthermore, introducing the methyl substit­uent into the benzene­sulfonyl ring of (I) to form (III) reduces the three-dimensional grid-like architecture into a one-dimensional ribbon architecture. However, in (III)–(VII), the introduction of a methyl substituent into the benzene­sulfonyl ring results in no change to the supra­molecular architecture.

## Synthesis and crystallization   

Compounds (I) and (II) were prepared by refluxing a mixture of 3-fluoro­benzoic acid, the corresponding substituted benzene­sulfonamides and phospho­rus oxychloride for 3 h on a water bath. The resultant mixtures were cooled and poured into ice-cold water. The solids obtained were filtered, washed thoroughly with water and then dissolved in sodium bicarbonate solutions. The compounds were later reprecipitated by acidifying the filtered solutions with dilute HCl. They were filtered, dried and recrystallized; m.p = 442–444 K for (I) and 422–423 K for (II). Prism-like, colourless single crystals of (I) and (II) were obtained by slow evaporation of the respective solutions of the compounds in methanol (with a few drops of water).

## Refinement   

Crystal data, data collection and structure refinement details are summarized in Table 3 ▸. The H atoms of the NH groups in (I) and (II) were located in a difference map and later refined freely. The other H atoms were positioned with idealized geometry using a riding model with C—H = 0.93–0.96 Å, and with *U*_iso_ = 1.2 or 1.5*U*_eq_(parent atom). To improve considerably the values of *R*1, *wR*2 and GOOF, reflections with very bad agreement (−20 0 0), (−20 0 10) and (−19 1 15) in (I) and (0 6 0) in (II) were omitted from the final refinements.

## Supplementary Material

Crystal structure: contains datablock(s) I, II, global. DOI: 10.1107/S2056989016003248/hb7565sup1.cif

Structure factors: contains datablock(s) I. DOI: 10.1107/S2056989016003248/hb7565Isup2.hkl

Structure factors: contains datablock(s) II. DOI: 10.1107/S2056989016003248/hb7565IIsup3.hkl

Supporting information file. DOI: 10.1107/S2056989016003248/hb7565Isup4.cml

Supporting information file. DOI: 10.1107/S2056989016003248/hb7565IIsup5.cml

CCDC references: 1418689, 1418688

Additional supporting information:  crystallographic information; 3D view; checkCIF report

## Figures and Tables

**Table 1:** Hydrogen-bond geometry (Å, °) for (I) *Cg*1 and *Cg*2 are the centroids of the sulfonyl and benzoyl rings, respectively.

*D*—H⋯*A*	*D*—H	H⋯*A*	*D*⋯*A*	*D*—H⋯*A*
N1—H1⋯O1^i^	0.81 (3)	2.08 (3)	2.883 (2)	171 (3)
C9—H9⋯O1^i^	0.93	2.42	3.244 (3)	148
C6—H6⋯O3^ii^	0.93	2.50	3.294 (3)	143
C2—H2⋯*Cg*1^iii^	0.93	2.82	3.474 (2)	129
C4—H4⋯*Cg*2^iv^	0.93	2.84	3.582 (2)	137
C11—H11⋯*Cg*1^v^	0.93	2.97	3.756 (3)	143

Symmetry codes: (i) 
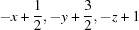
; (ii) 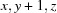; (iii) 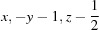; (iv) 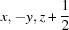; (v) 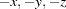.

**Table 2:** Hydrogen-bond geometry (Å, °) for (II)

*D*—H⋯*A*	*D*—H	H⋯*A*	*D*⋯*A*	*D*—H⋯*A*
N1—H1⋯O1^i^	0.87 (4)	2.06 (4)	2.937 (3)	177 (3)
C5—H5⋯O3^ii^	0.93	2.46	3.375 (3)	168
C13—H13⋯O1^i^	0.93	2.47	3.285 (3)	147

Symmetry codes: (i) 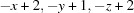; (ii) 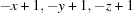.

**Table 3:** Experimental details

	(I)	(II)
Crystal data		
Chemical formula	C_13_H_10_FNO_3_S	C_14_H_12_FNO_3_S
*M*_r_	279.28	293.31
Crystal system, space group	Monoclinic, *C*2/*c*	Monoclinic, *P*2_1_/*c*
Temperature (K)	173	173
*a*, *b*, *c* (Å)	21.4036 (8), 5.7673 (2), 19.5525 (7)	9.0376 (4), 12.2912 (5), 12.1377 (5)
β (°)	92.135 (1)	105.107 (2)
*V* (Å^3^)	2411.90 (15)	1301.70 (9)
*Z*	8	4
Radiation type	Cu *K*α	Cu *K*α
μ (mm^−1^)	2.56	2.40
Crystal size (mm)	0.28 × 0.24 × 0.19	0.28 × 0.22 × 0.18

Data collection		
Diffractometer	Bruker APEXII	Bruker APEXII
Absorption correction	Multi-scan (*SADABS*; Bruker, 2009 ▸)	Multi-scan (*SADABS*; Bruker, 2009 ▸)
*T*_min_, *T*_max_	0.512, 0.614	0.557, 0.649
No. of measured, independent and observed [*I* > 2σ(*I*)] reflections	8647, 1985, 1846	8422, 2115, 1796
*R*_int_	0.037	0.056
(sin θ/λ)_max_ (Å^−1^)	0.587	0.583

Refinement		
*R*[*F* ^2^ > 2σ(*F* ^2^)], *wR*(*F* ^2^), *S*	0.042, 0.133, 0.98	0.050, 0.152, 1.05
No. of reflections	1985	2115
No. of parameters	176	186
H-atom treatment	H atoms treated by a mixture of independent and constrained refinement	H atoms treated by a mixture of independent and constrained refinement
Δρ_max_, Δρ_min_ (e Å^−3^)	0.39, −0.38	0.45, −0.47

Computer programs: *APEX2* and *SAINT-Plus* (Bruker, 2009 ▸), *SHELXS97* and *SHELXL97* (Sheldrick, 2008 ▸) and *Mercury* (Macrae *et al.*, 2008 ▸).

## supplementary crystallographic information

### (I) N-(3-Fluorobenzoyl)benzenesulfonamide Crystal data

**Table:**

C_13_H_10_FNO_3_S	Prism
*M**_r_* = 279.28	*D*_x_ = 1.538 Mg m^−^^3^
Monoclinic, *C*2/*c*	Melting point: 442 K
Hall symbol: -C 2yc	Cu *K*α radiation, λ = 1.54178 Å
*a* = 21.4036 (8) Å	Cell parameters from 123 reflections
*b* = 5.7673 (2) Å	θ = 4.1–64.8°
*c* = 19.5525 (7) Å	µ = 2.56 mm^−^^1^
β = 92.135 (1)°	*T* = 173 K
*V* = 2411.90 (15) Å^3^	Prism, colourless
*Z* = 8	0.28 × 0.24 × 0.19 mm
*F*(000) = 1152	

### (I) N-(3-Fluorobenzoyl)benzenesulfonamide Data collection

**Table:**

Bruker APEXII diffractometer	1846 reflections with *I* > 2σ(*I*)
Radiation source: fine-focus sealed tube	*R*_int_ = 0.037
Graphite monochromator	θ_max_ = 64.8°, θ_min_ = 4.1°
phi and φ scans	*h* = −24→24
Absorption correction: multi-scan (SADABS; Bruker, 2009)	*k* = −6→5
*T*_min_ = 0.512, *T*_max_ = 0.614	*l* = −22→22
8647 measured reflections	1 standard reflections every 1 reflections
1985 independent reflections	intensity decay: 0.1%

### (I) N-(3-Fluorobenzoyl)benzenesulfonamide Refinement

**Table:**

Refinement on *F*^2^	Primary atom site location: structure-invariant direct methods
Least-squares matrix: full	Secondary atom site location: difference Fourier map
*R*[*F*^2^ > 2σ(*F*^2^)] = 0.042	Hydrogen site location: inferred from neighbouring sites
*wR*(*F*^2^) = 0.133	H atoms treated by a mixture of independent and constrained refinement
*S* = 0.98	*w* = 1/[σ^2^(*F*_o_^2^) + (0.105*P*)^2^ + 2.9874*P*] where *P* = (*F*_o_^2^ + 2*F*_c_^2^)/3
1985 reflections	(Δ/σ)_max_ < 0.001
176 parameters	Δρ_max_ = 0.39 e Å^−^^3^
0 restraints	Δρ_min_ = −0.38 e Å^−^^3^

### (I) N-(3-Fluorobenzoyl)benzenesulfonamide Special details

**Table:**

Geometry. All s.u.'s (except the s.u. in the dihedral angle between two l.s. planes) are estimated using the full covariance matrix. The cell s.u.'s are taken into account individually in the estimation of s.u.'s in distances, angles and torsion angles; correlations between s.u.'s in cell parameters are only used when they are defined by crystal symmetry. An approximate (isotropic) treatment of cell s.u.'s is used for estimating s.u.'s involving l.s. planes.
Refinement. Refinement of F^2^ against ALL reflections. The weighted R-factor wR and goodness of fit S are based on F^2^, conventional R-factors R are based on F, with F set to zero for negative F^2^. The threshold expression of F^2^ > 2σ(F^2^) is used only for calculating R-factors(gt) etc. and is not relevant to the choice of reflections for refinement. R-factors based on F^2^ are statistically about twice as large as those based on F, and R- factors based on ALL data will be even larger.

### (I) N-(3-Fluorobenzoyl)benzenesulfonamide Fractional atomic coordinates and isotropic or equivalent isotropic displacement parameters (Å^2^)

**Table:**

	*x*	*y*	*z*	*U*_iso_*/*U*_eq_	
S1	0.26504 (2)	0.47902 (9)	0.41244 (2)	0.0162 (2)	
F1	0.46178 (7)	0.8164 (3)	0.66939 (7)	0.0325 (4)	
O2	0.24567 (7)	0.2440 (3)	0.40478 (8)	0.0220 (4)	
O1	0.21912 (7)	0.6490 (3)	0.42975 (8)	0.0230 (4)	
O3	0.38206 (7)	0.2258 (3)	0.43259 (8)	0.0233 (4)	
N1	0.31971 (9)	0.5057 (3)	0.47405 (10)	0.0174 (4)	
C8	0.41781 (9)	0.4102 (4)	0.53566 (11)	0.0175 (5)	
C7	0.37286 (10)	0.3690 (3)	0.47665 (11)	0.0176 (5)	
C3	0.33096 (10)	0.4990 (4)	0.22344 (12)	0.0205 (5)	
H3	0.3305	0.4048	0.1848	0.025*	
C9	0.41783 (10)	0.6094 (4)	0.57623 (11)	0.0195 (5)	
H9	0.3885	0.7264	0.5684	0.023*	
C5	0.36070 (10)	0.8565 (4)	0.27931 (12)	0.0215 (5)	
H5	0.3808	0.9994	0.2782	0.026*	
C2	0.30217 (10)	0.4254 (4)	0.28191 (11)	0.0180 (5)	
H2	0.2828	0.2811	0.2831	0.022*	
C6	0.33094 (9)	0.7874 (4)	0.33818 (11)	0.0188 (5)	
H6	0.3300	0.8840	0.3762	0.023*	
C10	0.46279 (10)	0.6266 (4)	0.62832 (11)	0.0215 (5)	
C1	0.30264 (9)	0.5700 (4)	0.33854 (10)	0.0153 (5)	
C4	0.36042 (10)	0.7129 (4)	0.22245 (12)	0.0217 (5)	
H4	0.3802	0.7605	0.1833	0.026*	
C11	0.50814 (11)	0.4618 (4)	0.64123 (12)	0.0247 (5)	
H11	0.5383	0.4820	0.6762	0.030*	
C13	0.46262 (10)	0.2394 (4)	0.54849 (12)	0.0228 (5)	
H13	0.4623	0.1061	0.5217	0.027*	
C12	0.50767 (11)	0.2653 (4)	0.60072 (13)	0.0268 (6)	
H12	0.5376	0.1504	0.6085	0.032*	
H1	0.3091 (14)	0.591 (5)	0.5042 (17)	0.035 (8)*	

### (I) N-(3-Fluorobenzoyl)benzenesulfonamide Atomic displacement parameters (Å^2^)

**Table:**

	*U*^11^	*U*^22^	*U*^33^	*U*^12^	*U*^13^	*U*^23^
S1	0.0155 (4)	0.0201 (4)	0.0130 (3)	0.00064 (18)	−0.0003 (2)	−0.00239 (18)
F1	0.0361 (8)	0.0312 (8)	0.0295 (8)	−0.0004 (6)	−0.0089 (6)	−0.0093 (6)
O2	0.0238 (8)	0.0243 (9)	0.0178 (8)	−0.0059 (7)	−0.0004 (6)	0.0001 (6)
O1	0.0178 (8)	0.0339 (9)	0.0173 (8)	0.0065 (7)	−0.0013 (6)	−0.0070 (6)
O3	0.0233 (8)	0.0233 (8)	0.0233 (9)	0.0036 (6)	0.0000 (6)	−0.0069 (7)
N1	0.0185 (10)	0.0213 (10)	0.0124 (9)	0.0031 (7)	−0.0009 (8)	−0.0035 (7)
C8	0.0152 (10)	0.0203 (11)	0.0171 (11)	0.0004 (9)	0.0026 (8)	0.0027 (9)
C7	0.0187 (10)	0.0160 (11)	0.0183 (11)	−0.0003 (8)	0.0029 (8)	0.0014 (8)
C3	0.0194 (11)	0.0257 (12)	0.0165 (11)	0.0020 (8)	0.0009 (9)	−0.0024 (8)
C9	0.0170 (10)	0.0211 (11)	0.0205 (11)	0.0006 (8)	0.0015 (8)	0.0021 (9)
C5	0.0194 (11)	0.0164 (11)	0.0286 (12)	0.0014 (8)	−0.0020 (9)	0.0040 (9)
C2	0.0182 (11)	0.0167 (10)	0.0190 (11)	0.0022 (9)	−0.0024 (8)	−0.0022 (9)
C6	0.0183 (10)	0.0172 (11)	0.0208 (11)	0.0026 (8)	−0.0023 (8)	−0.0026 (9)
C10	0.0224 (11)	0.0218 (12)	0.0203 (11)	−0.0042 (9)	0.0008 (9)	−0.0006 (9)
C1	0.0132 (10)	0.0178 (10)	0.0149 (10)	0.0041 (8)	−0.0007 (8)	0.0006 (9)
C4	0.0166 (10)	0.0281 (12)	0.0207 (12)	0.0027 (9)	0.0018 (8)	0.0084 (9)
C11	0.0172 (11)	0.0336 (13)	0.0229 (12)	−0.0031 (9)	−0.0037 (9)	0.0070 (10)
C13	0.0193 (11)	0.0223 (11)	0.0268 (12)	0.0024 (9)	0.0020 (9)	0.0001 (9)
C12	0.0189 (11)	0.0288 (13)	0.0323 (13)	0.0066 (9)	−0.0032 (9)	0.0041 (10)

### (I) N-(3-Fluorobenzoyl)benzenesulfonamide Geometric parameters (Å, º)

**Table:**

S1—O2	1.4238 (16)	C9—H9	0.9300
S1—O1	1.4375 (16)	C5—C4	1.386 (3)
S1—N1	1.6549 (19)	C5—C6	1.394 (3)
S1—C1	1.760 (2)	C5—H5	0.9300
F1—C10	1.358 (3)	C2—C1	1.386 (3)
O3—C7	1.215 (3)	C2—H2	0.9300
N1—C7	1.383 (3)	C6—C1	1.392 (3)
N1—H1	0.81 (3)	C6—H6	0.9300
C8—C13	1.391 (3)	C10—C11	1.375 (3)
C8—C9	1.396 (3)	C4—H4	0.9300
C8—C7	1.494 (3)	C11—C12	1.383 (4)
C3—C2	1.385 (3)	C11—H11	0.9300
C3—C4	1.386 (3)	C13—C12	1.386 (3)
C3—H3	0.9300	C13—H13	0.9300
C9—C10	1.378 (3)	C12—H12	0.9300
			
O2—S1—O1	118.36 (9)	C3—C2—C1	119.0 (2)
O2—S1—N1	111.12 (9)	C3—C2—H2	120.5
O1—S1—N1	103.67 (9)	C1—C2—H2	120.5
O2—S1—C1	109.75 (10)	C1—C6—C5	118.3 (2)
O1—S1—C1	109.12 (10)	C1—C6—H6	120.9
N1—S1—C1	103.73 (9)	C5—C6—H6	120.9
C7—N1—S1	122.13 (16)	F1—C10—C11	118.4 (2)
C7—N1—H1	125 (2)	F1—C10—C9	117.97 (19)
S1—N1—H1	112 (2)	C11—C10—C9	123.7 (2)
C13—C8—C9	119.6 (2)	C2—C1—C6	121.9 (2)
C13—C8—C7	116.5 (2)	C2—C1—S1	119.10 (17)
C9—C8—C7	123.80 (19)	C6—C1—S1	119.01 (16)
O3—C7—N1	121.08 (19)	C3—C4—C5	120.6 (2)
O3—C7—C8	122.62 (19)	C3—C4—H4	119.7
N1—C7—C8	116.29 (18)	C5—C4—H4	119.7
C2—C3—C4	120.1 (2)	C10—C11—C12	118.1 (2)
C2—C3—H3	120.0	C10—C11—H11	120.9
C4—C3—H3	120.0	C12—C11—H11	120.9
C10—C9—C8	117.7 (2)	C8—C13—C12	120.8 (2)
C10—C9—H9	121.2	C8—C13—H13	119.6
C8—C9—H9	121.2	C12—C13—H13	119.6
C4—C5—C6	120.2 (2)	C11—C12—C13	120.0 (2)
C4—C5—H5	119.9	C11—C12—H12	120.0
C6—C5—H5	119.9	C13—C12—H12	120.0
			
O2—S1—N1—C7	51.25 (19)	C5—C6—C1—C2	−1.5 (3)
O1—S1—N1—C7	179.42 (17)	C5—C6—C1—S1	179.79 (15)
C1—S1—N1—C7	−66.61 (19)	O2—S1—C1—C2	7.79 (19)
S1—N1—C7—O3	1.5 (3)	O1—S1—C1—C2	−123.41 (16)
S1—N1—C7—C8	−179.26 (14)	N1—S1—C1—C2	126.59 (16)
C13—C8—C7—O3	−16.3 (3)	O2—S1—C1—C6	−173.49 (15)
C9—C8—C7—O3	162.1 (2)	O1—S1—C1—C6	55.31 (18)
C13—C8—C7—N1	164.44 (19)	N1—S1—C1—C6	−54.69 (18)
C9—C8—C7—N1	−17.2 (3)	C2—C3—C4—C5	−0.9 (3)
C13—C8—C9—C10	−0.5 (3)	C6—C5—C4—C3	−0.3 (3)
C7—C8—C9—C10	−178.80 (19)	F1—C10—C11—C12	177.9 (2)
C4—C3—C2—C1	0.9 (3)	C9—C10—C11—C12	−1.6 (4)
C4—C5—C6—C1	1.5 (3)	C9—C8—C13—C12	−0.6 (3)
C8—C9—C10—F1	−177.86 (19)	C7—C8—C13—C12	177.9 (2)
C8—C9—C10—C11	1.6 (3)	C10—C11—C12—C13	0.5 (4)
C3—C2—C1—C6	0.3 (3)	C8—C13—C12—C11	0.6 (4)
C3—C2—C1—S1	179.00 (16)		

### (I) N-(3-Fluorobenzoyl)benzenesulfonamide Hydrogen-bond geometry (Å, º)

Cg1 and Cg2 are the centroids of the sulfonyl and benzoyl rings, respectively.

**Table:**

*D*—H···*A*	*D*—H	H···*A*	*D*···*A*	*D*—H···*A*
N1—H1···O1^i^	0.81 (3)	2.08 (3)	2.883 (2)	171 (3)
C9—H9···O1^i^	0.93	2.42	3.244 (3)	148
C6—H6···O3^ii^	0.93	2.50	3.294 (3)	143
C2—H2···*Cg*1^iii^	0.93	2.82	3.474 (2)	129
C4—H4···*Cg*2^iv^	0.93	2.84	3.582 (2)	137
C11—H11···*Cg*1^v^	0.93	2.97	3.756 (3)	143

Symmetry codes: (i) −*x*+1/2, −*y*+3/2, −*z*+1; (ii) *x*, *y*+1, *z*; (iii) *x*, −*y*−1, *z*−1/2; (iv) *x*, −*y*, *z*+1/2; (v) −*x*, −*y*, −*z*.

### (II) N-(3-Fluorobenzoyl)-4-methylbenzenesulfonamide Crystal data

**Table:**

C_14_H_12_FNO_3_S	Prism
*M**_r_* = 293.31	*D*_x_ = 1.497 Mg m^−^^3^
Monoclinic, *P*2_1_/*c*	Melting point: 423 K
Hall symbol: -P 2ybc	Cu *K*α radiation, λ = 1.54178 Å
*a* = 9.0376 (4) Å	Cell parameters from 142 reflections
*b* = 12.2912 (5) Å	θ = 5.1–64.1°
*c* = 12.1377 (5) Å	µ = 2.40 mm^−^^1^
β = 105.107 (2)°	*T* = 173 K
*V* = 1301.70 (9) Å^3^	Prism, colourless
*Z* = 4	0.28 × 0.22 × 0.18 mm
*F*(000) = 608	

### (II) N-(3-Fluorobenzoyl)-4-methylbenzenesulfonamide Data collection

**Table:**

Bruker APEXII diffractometer	1796 reflections with *I* > 2σ(*I*)
Radiation source: fine-focus sealed tube	*R*_int_ = 0.056
Graphite monochromator	θ_max_ = 64.1°, θ_min_ = 5.1°
phi and φ scans	*h* = −10→10
Absorption correction: multi-scan (SADABS; Bruker, 2009)	*k* = −14→14
*T*_min_ = 0.557, *T*_max_ = 0.649	*l* = −10→13
8422 measured reflections	1 standard reflections every 1 reflections
2115 independent reflections	intensity decay: 0.1%

### (II) N-(3-Fluorobenzoyl)-4-methylbenzenesulfonamide Refinement

**Table:**

Refinement on *F*^2^	Primary atom site location: structure-invariant direct methods
Least-squares matrix: full	Secondary atom site location: difference Fourier map
*R*[*F*^2^ > 2σ(*F*^2^)] = 0.050	Hydrogen site location: inferred from neighbouring sites
*wR*(*F*^2^) = 0.152	H atoms treated by a mixture of independent and constrained refinement
*S* = 1.05	*w* = 1/[σ^2^(*F*_o_^2^) + (0.1104*P*)^2^] where *P* = (*F*_o_^2^ + 2*F*_c_^2^)/3
2115 reflections	(Δ/σ)_max_ = 0.023
186 parameters	Δρ_max_ = 0.45 e Å^−^^3^
0 restraints	Δρ_min_ = −0.47 e Å^−^^3^

### (II) N-(3-Fluorobenzoyl)-4-methylbenzenesulfonamide Special details

**Table:**

Geometry. All s.u.'s (except the s.u. in the dihedral angle between two l.s. planes) are estimated using the full covariance matrix. The cell s.u.'s are taken into account individually in the estimation of s.u.'s in distances, angles and torsion angles; correlations between s.u.'s in cell parameters are only used when they are defined by crystal symmetry. An approximate (isotropic) treatment of cell s.u.'s is used for estimating s.u.'s involving l.s. planes.
Refinement. Refinement of F^2^ against ALL reflections. The weighted R-factor wR and goodness of fit S are based on F^2^, conventional R-factors R are based on F, with F set to zero for negative F^2^. The threshold expression of F^2^ > 2σ(F^2^) is used only for calculating R-factors(gt) etc. and is not relevant to the choice of reflections for refinement. R-factors based on F^2^ are statistically about twice as large as those based on F, and R- factors based on ALL data will be even larger.

### (II) N-(3-Fluorobenzoyl)-4-methylbenzenesulfonamide Fractional atomic coordinates and isotropic or equivalent isotropic displacement parameters (Å^2^)

**Table:**

	*x*	*y*	*z*	*U*_iso_*/*U*_eq_	
S1	0.86791 (7)	0.55562 (5)	0.81354 (5)	0.0171 (3)	
F1	0.17463 (17)	0.30702 (14)	0.94022 (13)	0.0303 (4)	
O1	1.0242 (2)	0.55041 (16)	0.88113 (15)	0.0231 (5)	
O2	0.7956 (2)	0.65901 (15)	0.79047 (14)	0.0240 (5)	
O3	0.5443 (2)	0.48889 (17)	0.75791 (15)	0.0260 (5)	
N1	0.7768 (2)	0.47863 (18)	0.88728 (19)	0.0183 (5)	
C8	0.5545 (3)	0.3863 (2)	0.9263 (2)	0.0169 (5)	
C2	0.9568 (3)	0.4058 (2)	0.6775 (2)	0.0205 (6)	
H2	1.0292	0.3827	0.7426	0.025*	
C9	0.3950 (3)	0.3781 (2)	0.8985 (2)	0.0199 (6)	
H9	0.3346	0.4151	0.8360	0.024*	
C13	0.6429 (3)	0.3294 (2)	1.0197 (2)	0.0192 (6)	
H13	0.7492	0.3351	1.0387	0.023*	
C12	0.5722 (3)	0.2640 (2)	1.0844 (2)	0.0206 (6)	
H12	0.6318	0.2251	1.1457	0.025*	
C5	0.7362 (3)	0.4729 (2)	0.4840 (2)	0.0214 (6)	
H5	0.6619	0.4947	0.4193	0.026*	
C3	0.9501 (3)	0.3593 (2)	0.5727 (2)	0.0204 (6)	
H3	1.0200	0.3053	0.5672	0.024*	
C10	0.3296 (3)	0.3141 (2)	0.9655 (2)	0.0207 (6)	
C4	0.8399 (3)	0.3922 (2)	0.4747 (2)	0.0210 (6)	
C11	0.4148 (3)	0.2562 (2)	1.0587 (2)	0.0212 (6)	
H11	0.3671	0.2134	1.1026	0.025*	
C6	0.7415 (3)	0.5214 (2)	0.5878 (2)	0.0189 (6)	
H6	0.6718	0.5754	0.5931	0.023*	
C7	0.6213 (3)	0.4561 (2)	0.8499 (2)	0.0191 (6)	
C1	0.8532 (3)	0.4879 (2)	0.6840 (2)	0.0169 (6)	
C14	0.8314 (4)	0.3399 (2)	0.3606 (2)	0.0299 (7)	
H14A	0.7802	0.2710	0.3563	0.045*	
H14B	0.9331	0.3291	0.3524	0.045*	
H14C	0.7754	0.3864	0.3006	0.045*	
H1	0.833 (4)	0.469 (3)	0.957 (3)	0.033 (9)*	

### (II) N-(3-Fluorobenzoyl)-4-methylbenzenesulfonamide Atomic displacement parameters (Å^2^)

**Table:**

	*U*^11^	*U*^22^	*U*^33^	*U*^12^	*U*^13^	*U*^23^
S1	0.0170 (4)	0.0203 (4)	0.0131 (4)	−0.0036 (2)	0.0024 (3)	−0.0003 (2)
F1	0.0154 (8)	0.0438 (11)	0.0311 (9)	−0.0039 (7)	0.0050 (6)	0.0043 (7)
O1	0.0181 (10)	0.0340 (12)	0.0153 (9)	−0.0082 (8)	0.0011 (7)	−0.0010 (7)
O2	0.0298 (10)	0.0223 (10)	0.0204 (10)	−0.0003 (8)	0.0075 (8)	−0.0008 (7)
O3	0.0173 (10)	0.0386 (12)	0.0184 (10)	−0.0005 (8)	−0.0019 (8)	0.0092 (8)
N1	0.0152 (11)	0.0245 (12)	0.0127 (11)	−0.0033 (9)	−0.0006 (9)	0.0027 (9)
C8	0.0159 (12)	0.0170 (13)	0.0172 (12)	−0.0003 (10)	0.0032 (9)	−0.0031 (10)
C2	0.0161 (12)	0.0251 (15)	0.0181 (13)	−0.0024 (11)	0.0004 (10)	0.0029 (10)
C9	0.0175 (12)	0.0233 (14)	0.0169 (13)	0.0008 (11)	0.0012 (10)	0.0015 (10)
C13	0.0164 (12)	0.0210 (14)	0.0192 (13)	0.0010 (10)	0.0030 (10)	−0.0021 (10)
C12	0.0250 (13)	0.0183 (14)	0.0167 (12)	0.0011 (11)	0.0020 (10)	0.0015 (10)
C5	0.0216 (13)	0.0255 (14)	0.0141 (13)	−0.0027 (11)	−0.0005 (10)	0.0027 (10)
C3	0.0204 (13)	0.0188 (13)	0.0227 (13)	−0.0006 (11)	0.0070 (10)	0.0005 (10)
C10	0.0128 (12)	0.0273 (15)	0.0215 (13)	−0.0009 (10)	0.0035 (10)	−0.0044 (10)
C4	0.0242 (14)	0.0212 (14)	0.0186 (13)	−0.0070 (11)	0.0074 (10)	−0.0004 (10)
C11	0.0252 (13)	0.0207 (14)	0.0201 (13)	−0.0025 (11)	0.0101 (11)	−0.0010 (10)
C6	0.0183 (13)	0.0199 (13)	0.0170 (13)	−0.0014 (11)	0.0020 (10)	0.0008 (10)
C7	0.0176 (13)	0.0224 (15)	0.0173 (13)	−0.0005 (11)	0.0048 (11)	−0.0018 (10)
C1	0.0165 (13)	0.0187 (13)	0.0161 (13)	−0.0044 (10)	0.0054 (10)	0.0001 (10)
C14	0.0428 (17)	0.0295 (16)	0.0189 (13)	−0.0069 (13)	0.0103 (12)	−0.0024 (11)

### (II) N-(3-Fluorobenzoyl)-4-methylbenzenesulfonamide Geometric parameters (Å, º)

**Table:**

S1—O2	1.423 (2)	C13—H13	0.9300
S1—O1	1.4383 (19)	C12—C11	1.378 (4)
S1—N1	1.661 (2)	C12—H12	0.9300
S1—C1	1.753 (2)	C5—C6	1.383 (4)
F1—C10	1.356 (3)	C5—C4	1.389 (4)
O3—C7	1.220 (3)	C5—H5	0.9300
N1—C7	1.387 (3)	C3—C4	1.398 (4)
N1—H1	0.87 (4)	C3—H3	0.9300
C8—C13	1.393 (4)	C10—C11	1.388 (4)
C8—C9	1.396 (4)	C4—C14	1.510 (4)
C8—C7	1.500 (4)	C11—H11	0.9300
C2—C3	1.382 (4)	C6—C1	1.392 (4)
C2—C1	1.393 (4)	C6—H6	0.9300
C2—H2	0.9300	C14—H14A	0.9600
C9—C10	1.371 (4)	C14—H14B	0.9600
C9—H9	0.9300	C14—H14C	0.9600
C13—C12	1.391 (4)		
			
O2—S1—O1	118.99 (11)	C2—C3—C4	120.9 (2)
O2—S1—N1	110.36 (11)	C2—C3—H3	119.5
O1—S1—N1	102.63 (11)	C4—C3—H3	119.5
O2—S1—C1	108.91 (11)	F1—C10—C9	118.8 (2)
O1—S1—C1	108.91 (11)	F1—C10—C11	118.2 (2)
N1—S1—C1	106.26 (11)	C9—C10—C11	123.0 (2)
C7—N1—S1	122.65 (19)	C5—C4—C3	118.9 (2)
C7—N1—H1	125 (2)	C5—C4—C14	120.3 (2)
S1—N1—H1	111 (2)	C3—C4—C14	120.8 (2)
C13—C8—C9	119.8 (2)	C12—C11—C10	118.0 (2)
C13—C8—C7	123.5 (2)	C12—C11—H11	121.0
C9—C8—C7	116.7 (2)	C10—C11—H11	121.0
C3—C2—C1	118.9 (2)	C5—C6—C1	118.9 (2)
C3—C2—H2	120.5	C5—C6—H6	120.6
C1—C2—H2	120.5	C1—C6—H6	120.6
C10—C9—C8	118.5 (2)	O3—C7—N1	121.4 (2)
C10—C9—H9	120.8	O3—C7—C8	121.9 (2)
C8—C9—H9	120.8	N1—C7—C8	116.6 (2)
C12—C13—C8	119.9 (2)	C6—C1—C2	121.1 (2)
C12—C13—H13	120.0	C6—C1—S1	118.8 (2)
C8—C13—H13	120.0	C2—C1—S1	120.01 (19)
C11—C12—C13	120.8 (2)	C4—C14—H14A	109.5
C11—C12—H12	119.6	C4—C14—H14B	109.5
C13—C12—H12	119.6	H14A—C14—H14B	109.5
C6—C5—C4	121.2 (2)	C4—C14—H14C	109.5
C6—C5—H5	119.4	H14A—C14—H14C	109.5
C4—C5—H5	119.4	H14B—C14—H14C	109.5
			
O2—S1—N1—C7	55.4 (2)	C4—C5—C6—C1	−0.1 (4)
O1—S1—N1—C7	−176.8 (2)	S1—N1—C7—O3	2.7 (4)
C1—S1—N1—C7	−62.5 (2)	S1—N1—C7—C8	−179.31 (17)
C13—C8—C9—C10	0.6 (4)	C13—C8—C7—O3	166.5 (3)
C7—C8—C9—C10	179.3 (2)	C9—C8—C7—O3	−12.1 (4)
C9—C8—C13—C12	0.4 (4)	C13—C8—C7—N1	−11.5 (4)
C7—C8—C13—C12	−178.2 (2)	C9—C8—C7—N1	169.9 (2)
C8—C13—C12—C11	−1.3 (4)	C5—C6—C1—C2	−1.1 (4)
C1—C2—C3—C4	−1.2 (4)	C5—C6—C1—S1	175.79 (19)
C8—C9—C10—F1	179.0 (2)	C3—C2—C1—C6	1.7 (4)
C8—C9—C10—C11	−0.9 (4)	C3—C2—C1—S1	−175.14 (18)
C6—C5—C4—C3	0.6 (4)	O2—S1—C1—C6	−19.8 (2)
C6—C5—C4—C14	179.7 (2)	O1—S1—C1—C6	−150.97 (19)
C2—C3—C4—C5	0.0 (4)	N1—S1—C1—C6	99.1 (2)
C2—C3—C4—C14	−179.0 (2)	O2—S1—C1—C2	157.1 (2)
C13—C12—C11—C10	1.0 (4)	O1—S1—C1—C2	25.9 (2)
F1—C10—C11—C12	−179.7 (2)	N1—S1—C1—C2	−84.0 (2)
C9—C10—C11—C12	0.1 (4)		

### (II) N-(3-Fluorobenzoyl)-4-methylbenzenesulfonamide Hydrogen-bond geometry (Å, º)

**Table:**

*D*—H···*A*	*D*—H	H···*A*	*D*···*A*	*D*—H···*A*
N1—H1···O1^i^	0.87 (4)	2.06 (4)	2.937 (3)	177 (3)
C5—H5···O3^ii^	0.93	2.46	3.375 (3)	168
C13—H13···O1^i^	0.93	2.47	3.285 (3)	147

Symmetry codes: (i) −*x*+2, −*y*+1, −*z*+2; (ii) −*x*+1, −*y*+1, −*z*+1.

## References

[bb1] Banwell, M. G., Crasto, C. F., Easton, C. J., Forrest, A. K., Karoli, T., March, D. R., Mensah, L., Nairn, M. R., O’Hanlon, P. J., Oldham, M. D. & Yue, W. (2000). *Bioorg. Med. Chem. Lett.* **10**, 2263–2266. 10.1016/s0960-894x(00)00456-x 11055334

[bb2] Bruker (2009). *APEX2, *SADABS* and *SAINT-Plus*.* Bruker AXS Inc., Madison, Wisconsin, USA.

[bb3] Chang, L. L., Ashton, W. T., Flanagan, K. L., Chen, T. B., O’Malley, S. S., Zingaro, G. J., Siegl, P. K. S., Kivlighn, S. D., Lotti, V. J., Chang, R. S. L. & & Greenlee, W. J. (1994). *J. Med. Chem.* **37**, 4464–4478. 10.1021/jm00052a006 7799397

[bb4] Gowda, B. T., Foro, S., Suchetan, P. A. & Fuess, H. (2009*a*). *Acta Cryst.* E**65**, o2516. 10.1107/S1600536809037222 PMC2970249 21577963

[bb5] Gowda, B. T., Foro, S., Suchetan, P. A. & Fuess, H. (2009*b*). *Acta Cryst.* E**65**, o2750. 10.1107/S1600536809041051 PMC2971321 21578344

[bb6] Hasegawa, T. & Yamamoto, H. (2000). *Bull. Chem. Soc. Jpn*, **73**, 423–428.

[bb7] Macrae, C. F., Bruno, I. J., Chisholm, J. A., Edgington, P. R., McCabe, P., Pidcock, E., Rodriguez-Monge, L., Taylor, R., van de Streek, J. & Wood, P. A. (2008). *J. Appl. Cryst.* **41**, 466–470.

[bb8] Mader, M., Shih, C., Considine, E., Dios, A. D., Grossman, C., Hipskind, P., Lin, H., Lobb, K., Lopez, B., Lopez, J., Cabrejas, L., Richett, M., White, W., Cheung, Y., Huang, Z., Reilly, J. & Dinn, S. (2005). *Bioorg. Med. Chem. Lett.* **15**, 617–620. 10.1016/j.bmcl.2004.11.041 15664824

[bb9] Musser, J. H., Kreft, A. F., Bender, R. H. W., Kubrak, D. M., Grimes, D., Carlson, R. P., Hand, J. M. & Chang, J. (1990). *J. Med. Chem.* **33**, 240–245. 10.1021/jm00163a039 2104935

[bb10] Sheldrick, G. M. (2008). *Acta Cryst.* A**64**, 112–122. 10.1107/S0108767307043930 18156677

[bb11] Sreenivasa, S., Mohan, N. R., Manojkumar, K. E. & Suchetan, P. A. (2014). *J. Appl. Chem.* **3**, 551–559.

[bb12] Suchetan, P. A., Foro, S. & Gowda, B. T. (2012). *Acta Cryst.* E**68**, o1327. 10.1107/S1600536812013931 PMC3344466 22590228

[bb13] Suchetan, P. A., Gowda, B. T., Foro, S. & Fuess, H. (2010). *Acta Cryst.* E**66**, o1039. 10.1107/S1600536810011967 PMC2979201 21579100

